# Supplementary material for: A distinct mammalian disome collision interface harbors K63-linked polyubiquitination of uS10 to trigger hRQT-mediated subunit dissociation
Source: Nat Commun. 2022 Oct 27;13:6411. doi: 10.1038/s41467-022-34097-9 (PMC9613687; doi:10.1038/s41467-022-34097-9)
Supplement: Supplementary file 1 — Supplementary Information [file 41467_2022_34097_MOESM1_ESM.pdf]

Supplementary Information

A distinct mammalian disome collision interface harbors K63-linked polyubiquitination of uS10 to trigger hRQT-mediated subunit dissociation

Momoko Narita[1,2,#], Timo Denk[3,#], Yoshitaka Matsuo[1], Takato Sugiyama[2], Chisato Kikuguchi[1], Sota Ito[1], Nichika Sato[1], Toru Suzuki[1], Satoshi Hashimoto[2], Iva Machová[4], Petr Tesina[3], Roland Beckmann[3,*] and Toshifumi Inada[1,2,*]

[1]Division of RNA and gene regulation, Institute of Medical Science, The University of Tokyo, Minato-Ku 108-8639, Japan, [2]Graduate School of Pharmaceutical Sciences, Tohoku University, Sendai 980-8578, Japan, [3]Gene Center and Department of Biochemistry, University of Munich, Feodor-Lynen-Str. 25, 81377 Munich, Germany. [4]Biomedical Centre, Faculty of Medicine in Pilsen, Charles University in Prague, Alej Svobody 1655/76, 323 00 Pilsen, Czech Republic.

[#]These authors contributed equally to this study.

*Corresponding authors:
Prof. Dr. Roland Beckmann. Email: beckmann@genzentrum.lmu.de (R.B.)
Prof. Dr. Toshifumi Inada. Email: toshiinada@ims.u-tokyo.ac.jp (T.I.)

Lead contact:
To whom correspondence should be addressed: Toshifumi Inada, Minato-Ku 108-8639, Tokyo, Japan.
Tel: +81 (3)5449-5275, Fax: +81 (3)5449-5421, E-mail: toshiinada@ims.u-tokyo.ac.jp (T.I.)

Running title: hRQT dissociates colliding ribosome with K63-linked ubiquitin chain

This PDF file includes:
Supplementary Figures 1 to 6
Supplementary Tables 1 to 5

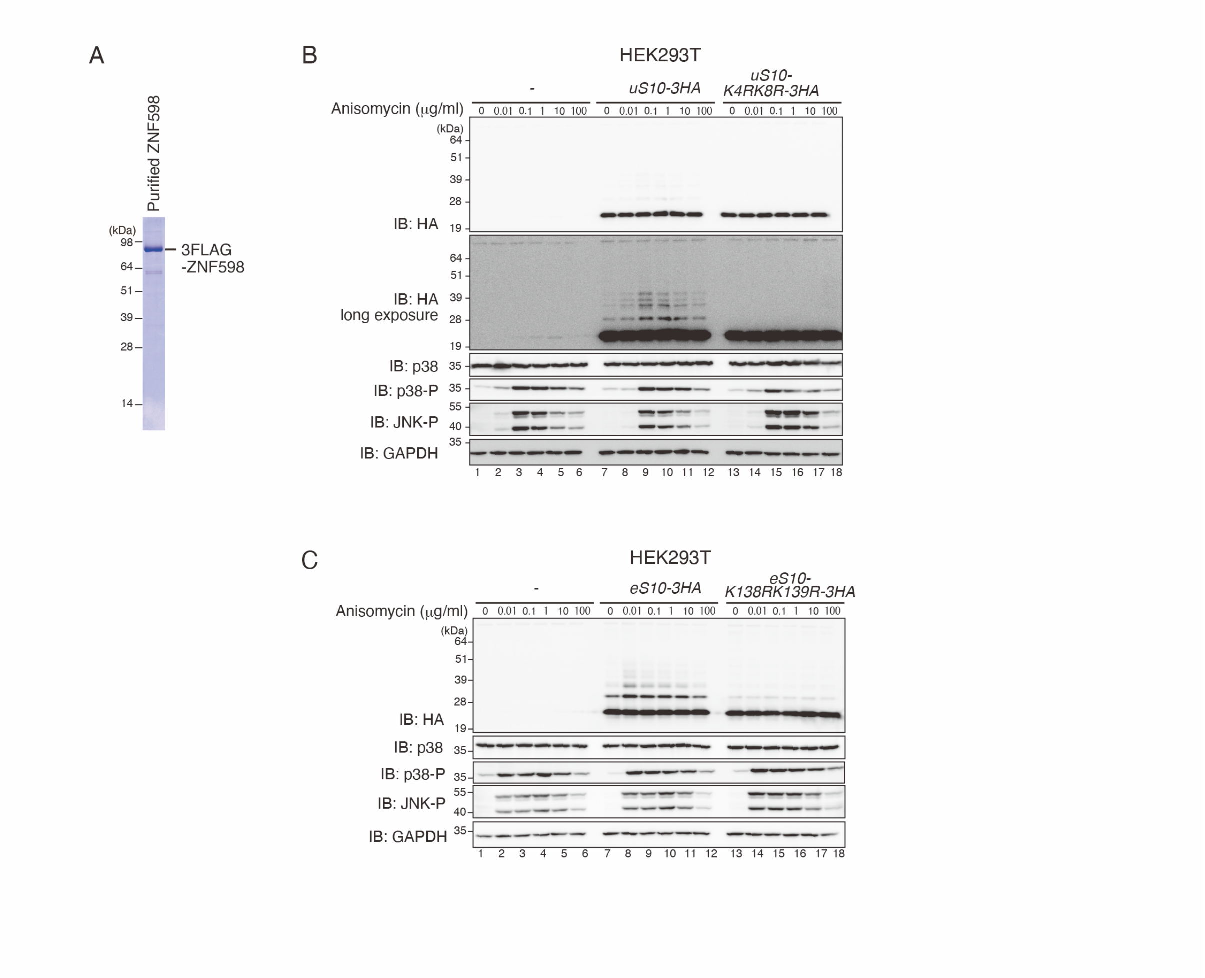

Supplementary Figure 1. Purification of ZNF598 and ribosome collision induction with anisomycin in HEK293T cells

(A) Purification of overexpressed 3×FLAG-ZNF598 from HEK293T cells. Purified protein was detected by Coomassie staining. **(B-C)** HEK293T cells expressing uS10-3HA **(B)**, uS10-K4RK8R-3HA or **(C)** eS10-3HA, eS10-K138RK139R-3HA and controls. Cells were treated with anisomycin at indicated concentrations to cause ribosome stalling *in vivo*. Cell lysates were prepared after 15 min incubation and were subjected to SDS-PAGE followed by Western Blotting with antibodies against HA, p38, phosphorylated form of p38, phosphorylated form of JNK and GAPDH. Elevation of p38 and JNK phosphorylation were used as a proxy to confirm ribosome collision induction [1]. The levels of ubiquitinated uS10-HA were correlated with the levels of the phosphorylated forms of p38 and JNK. These results are consistent with the previously proposed model in which ZAKα is self-activated by interacting with colliding ribosomes [1]. We obtained essentially the same results of at least five independent experiments.

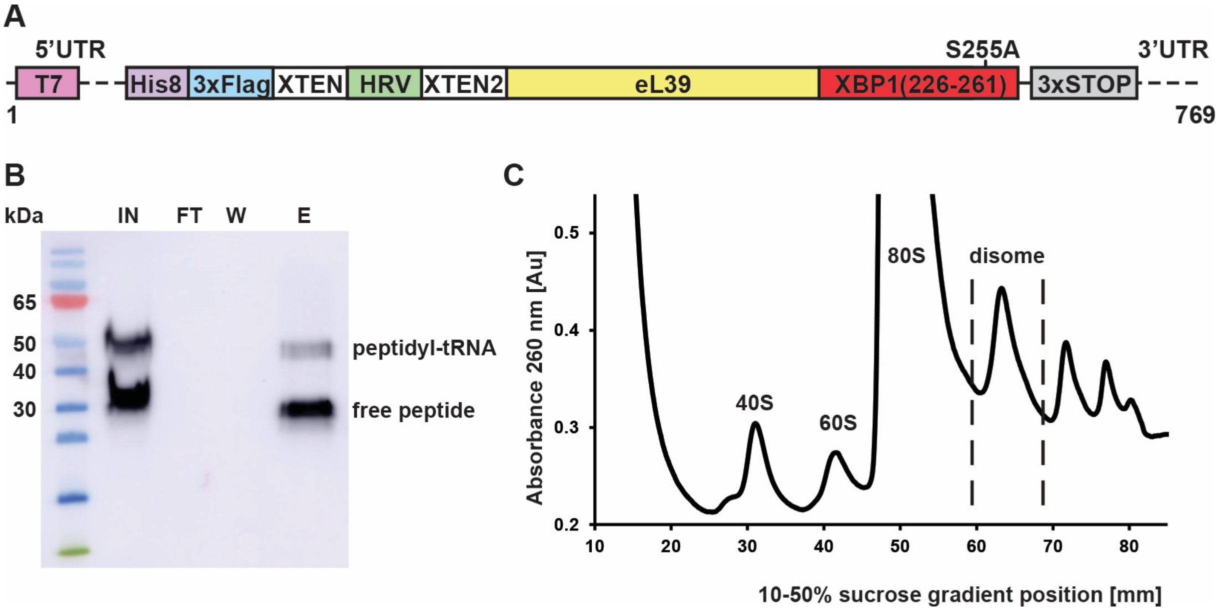

Supplementary Figure 2. Preparation of the human disome collided on the *Xbp1-XTEN* mRNA for cryo-EM analysis

(A) Schematic drawing of the *Xbp1-XTEN* mRNA used in the HeLa translation extract. A S255A modified XBP1u stalling sequence was inserted in an endogenous mRNA encoding eL39. An N-terminal tag sequence encodes for an HRV 3C protease cleavable His_8-tag for purification and a 3FLAG tag for immunoblotting. Accessibility of the cleavage site was enhanced by neutral disordered XTEN linkers [2]. **(B)** After the translation reaction, ribosome nascent chain complexes (RNCs) were purified using Dynabeads His-Tag Isolation and Pulldown beads (Invitrogen) for affinity purification of His-tagged nascent peptide. The beads were washed three times and eluted using imidazole. Fractions (1/100) representing input (IN), flow through (FT), washing step (W) and elution (E) were visualized by immunoblotting using an anti-Flag antibody. The presence of peptidyl-tRNA indicates ribosomal stalling. The gel represents a sample preparation experiment, which was performed three times with similar results. We obtained essentially the same results of at least three independent experiments. **(C)** The eluate was loaded on a 10-50 % sucrose gradient and fractionated. The disome peak was collected and ribosomes were concentrated by pelleting through a sucrose cushion. Resuspended pellets were used for cryo-EM.

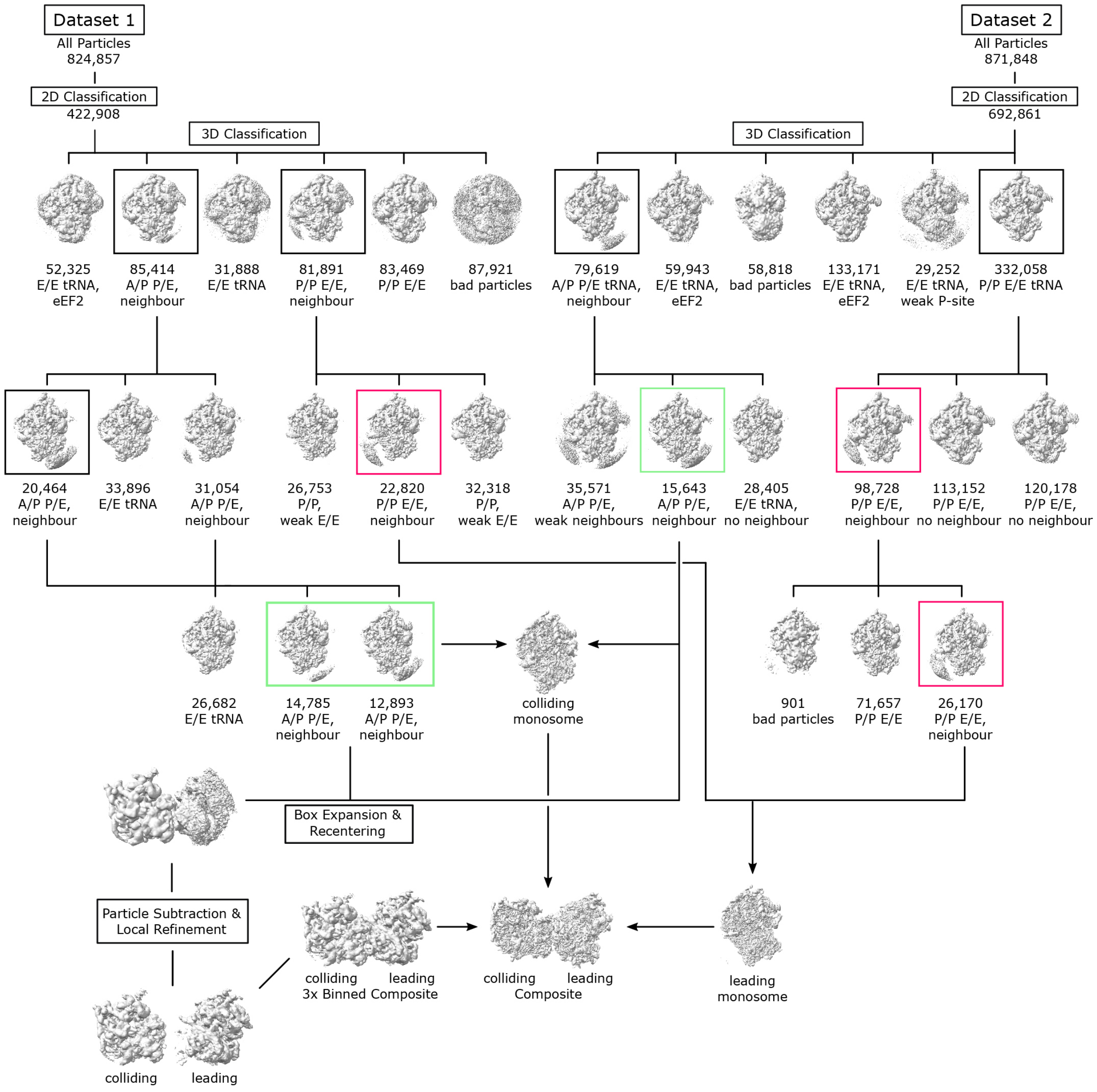

Supplementary Figure 3: Classification and processing scheme of cryo-EM data for the XBP1 disome

Two disome datasets were collected and initially processed separately. A total of 1,115,769 particles were selected after 2D classification. Initial refinements and 3D classifications into six classes were performed for each dataset. Numbers below each class represent particle counts. Classes in black rectangles were further processed. Classes in green rectangles represent the second colliding ribosome with extra density of the leading ribosome at the mRNA entry side. Classes in red rectangles represent the leading ribosome with an extra density of the colliding ribosome at the mRNA exit side. These classes were used for the final reconstructions and fitted into the consensus refinement density.

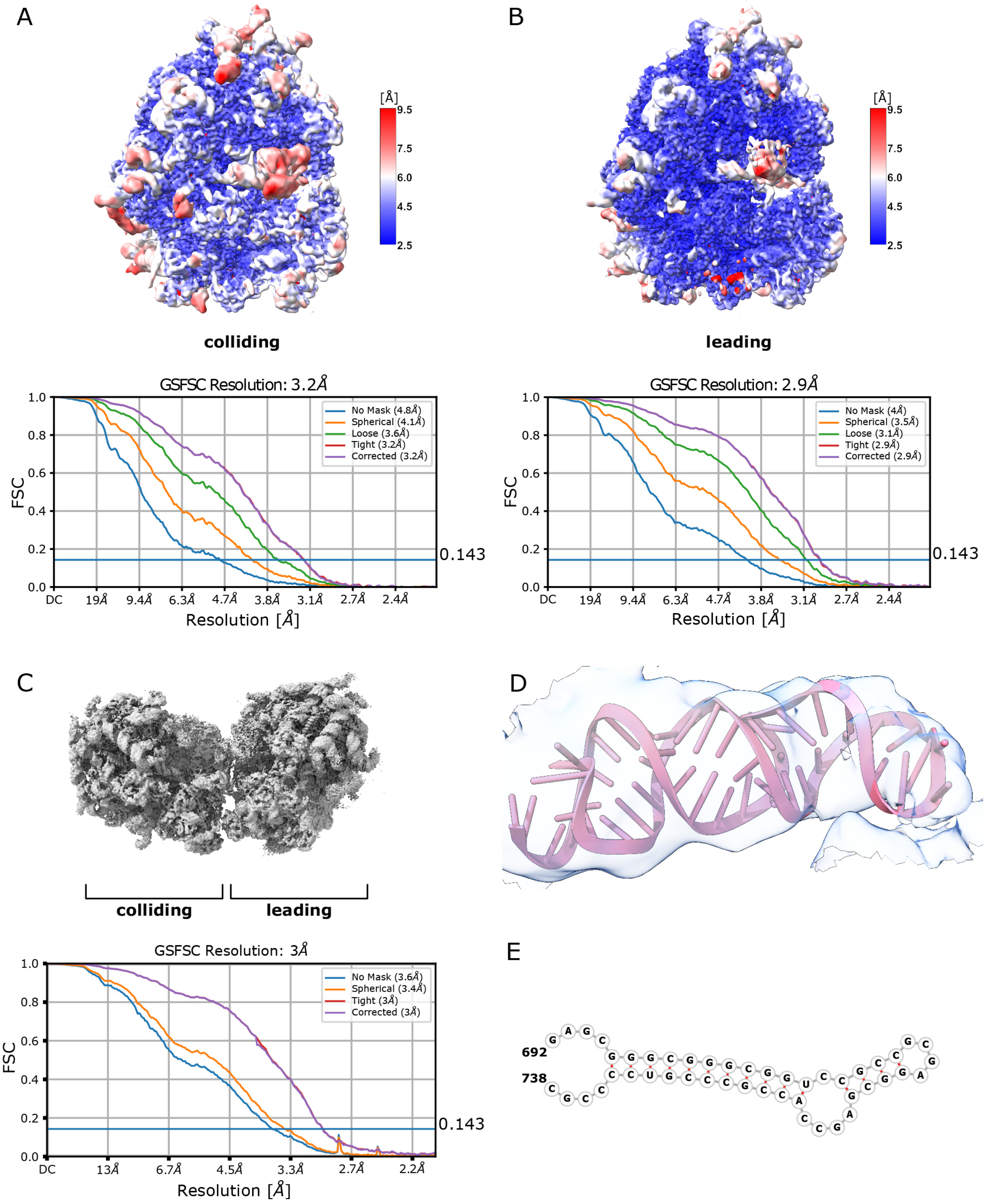

Supplementary Figure 4: Local resolution and Fourier shell correlation (FSC) curves for cryo-EM density maps, detail on es6c-2

Cryo-EM density maps were colored according to local resolution using cryoSPARC 3.2 [3] and visualized in UCSF ChimeraX 1.25 [4]. FSC curves were calculated in cryoSPARC 3.2. **(A)** Local resolution and FSC curves of the colliding ribosome. **(B)** Local resolution and FSC curves of the leading ribosome. **(C)** Composite map of the whole disome and corresponding FSC curves calculated using composite half-maps. **(D)** Cryo-EM density (transparent) and molecular model of es6c of the colliding ribosome (es6c-2). **(E)** Secondary structure prediction of the es6c-2 visualized in forna [5].

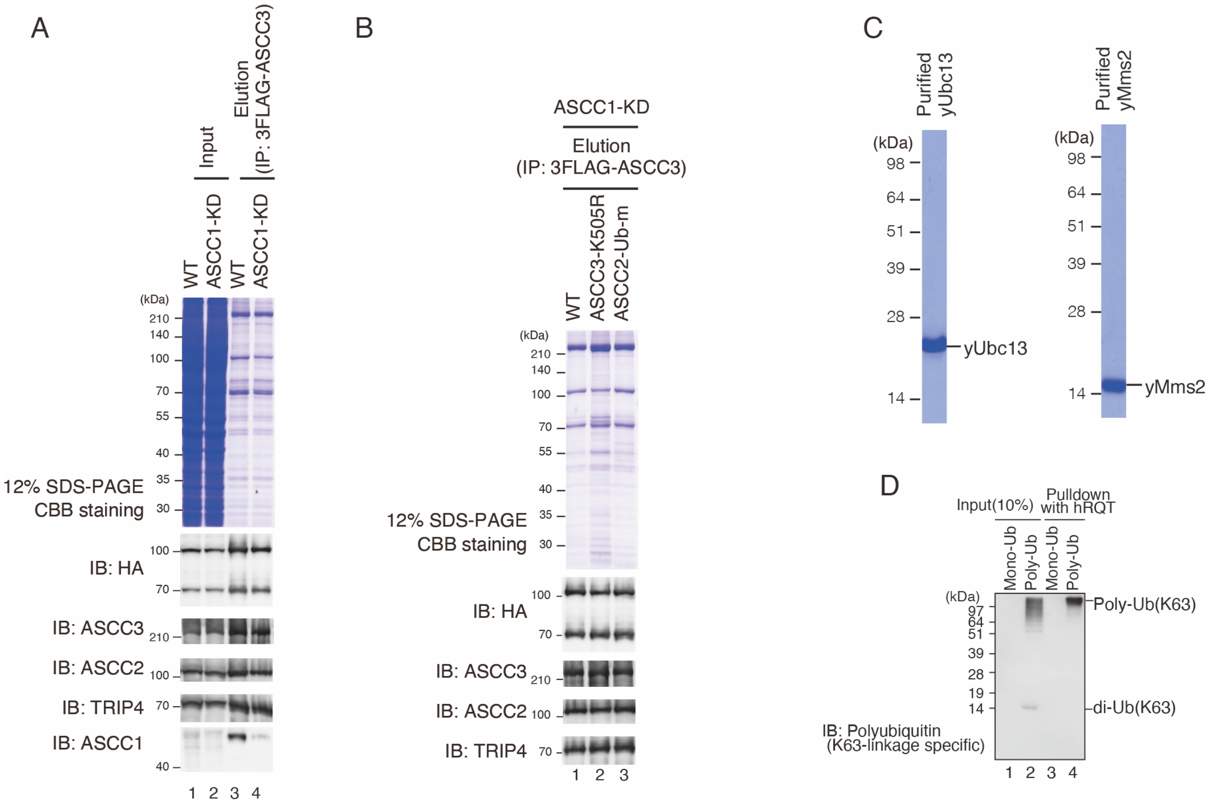

Supplementary Figure 5. Purification of the hRQT complex, the heterodimeric E2 ubiquitin-conjugating Ubc13–Mms2 complex and free K63-linked polyubiquitin chains

(A-B) The hRQT complex was purified from HEK293T cells. To exclude the ASCC1 protein which should not play a role in the cytoplasmic hRQT complex, ASCC1 was knocked down using shRNA. Indicated RQT factors were overexpressed including tagged 3×FLAG-ASCC3. Purified proteins were detected by Coomassie staining and Western Blotting. Purification of the wild-type **(A)** and the mutant **(B)** hRQT complex containing either ATPase deficient ASCC3-K505R or ubiquitin binding impaired ASCC2-Ubm. **(C)** Purification of E2 conjugating enzyme Ubc13 and the Mms2 protein from *E. coli*. Purified proteins were detected by Coomassie staining. **(D)** *In vitro* binding assay of K63-linked polyubiquitin chain with the hRQT complex. The K63-linked polyubiquitin chain associates with the hRQT complex. The ubiquitin proteins were incubated with ATP and the heterodimeric E2 ubiquitin-conjugating Ubc13-Mms2 complex. The hRQT complex composed of FLAG-ASCC3, HA-ASCC2 and HA-TRIP4 on the beads were incubated with monoubiquitin or K63-linked polyubiquitin chain**.** Samples were analysed by western blotting with anti-polyubiquitin (K63-linkage specific) antibody. We obtained essentially the same results of two independent experiments.

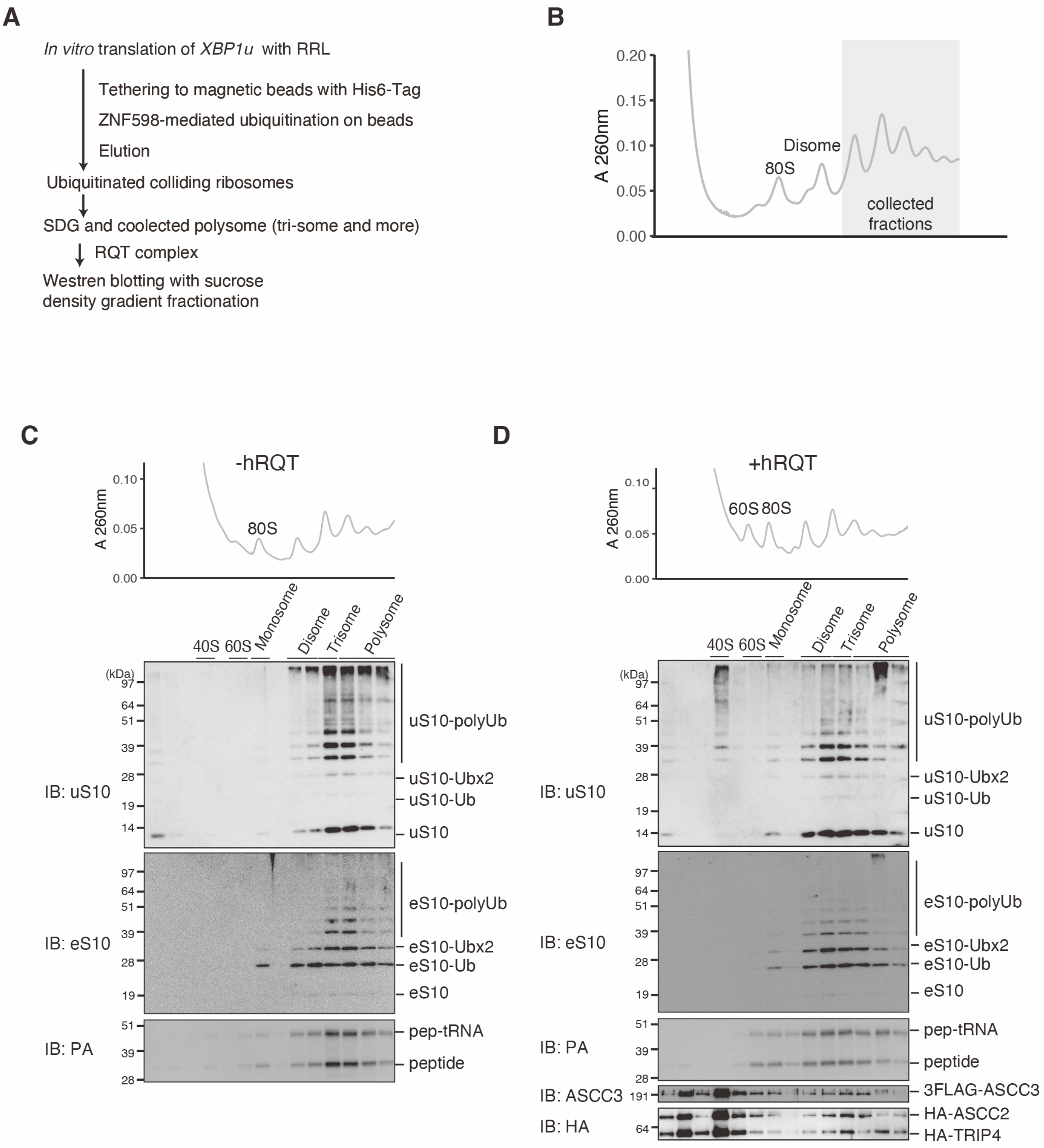

Supplementary Figure 6. The hRQT complex dissociates the purified collided ribosomes with K63-linked polyubiquitin chains on uS10.

(A) Schematic overview of hRQT complex-mediated subunit disassociation assay of the purified polyubiquitinated ribosomes using *XBP1u* stalled collided ribosomes. **(B)** Purified the ubiquitinated *XBP1u*-RNCs were separated by sucrose density gradient ultracentrifugation. Indicated polysome fractions (polysomes more than trisomes) were collected for the hRQT complex-mediated subunit disassociation assay. **(C-D)** The purified ubiquitinated RNCs were incubated without **(C)** or with **(D)** the hRQT complex. Ribosomal species resulting from the splitting reaction were separated by ultracentrifugation through sucrose density gradients. Gradient fractions were analysed by Western blotting using antibodies against ribosomal proteins uS10 and eS10 and against HA-tag and PA-tag and ASCC3. We obtained essentially the same results of at least three independent experiments.

	XBP1 disome<br>EMD- 14181<br>PDB 7QVP
**Data collection and processing**	
Magnification	130,000
Voltage (kV)	300
Electron exposure (e^{-}/Å^{2})	43.6
Defocus range (μm)	-0.4 – -3.5
Pixel size (Å)	1.045
Symmetry imposed	/
Initial particle images (no.)	1,115,769
Final particle images (no.)	53,848
Map resolution (Å)	3.0
FSC threshold	0.143
**Refinement**	
Initial models used (PDB codes)	6Y57, 6Y2L, 6Z6L
Model resolution (Å)	3.0
FSC threshold	0.5
Map sharpening *B* factor (Å^{2})	-1
Model composition	
Nonhydrogen atoms	417,877
Protein residues	22,169
Nucleotide residues	11,477
Ligands	366
mean *B* factors (Å^{2})	
Protein	113.66
Ligand	86.02
R.m.s. deviations	
Bond lengths (Å)	0.008
Bond angles (°)	0.993
**Validation**	
MolProbity score	1.87
Clashscore	7.16
Poor rotamers (%)	0.57
Ramachandran plot	
Favored (%)	92.25
Allowed (%)	7.55
Disallowed (%)	0.2

Supplementary Table 1: Cryo-EM data collection, refinement and validation statistics

Vector backbone	Inserted sequence	Reference
pcDNA3.1(+)	kozak-HA-XBP1u-V5	Han *et al.*, 2020
pcDNA3.1(+)	kozak-HA-XBP1u(W256A)-V5	This study
pcDNA3.1(+)	3FLAG-ZNF598	This study
pcDNA3.1(+)	3FLAG-ASCC3	Hashimoto *et al.*,2020
pcDNA3.1(+)	3FLAG-ASCC3 K505R	Hashimoto *et al.*,2020
pcDNA3.1(+)	HA-ASCC2	Hashimoto *et al.*,2020
pcDNA3.1(+)	HA-ASCC2 Ub-m	Hashimoto *et al.*,2020
pcDNA3.1(+)	HA-TRIP4	Hashimoto *et al.*,2020
pGEX6P1	GST-Ubc13	Saeki *et al.*, 2004
pGEX6P1	GST-Mms2	Saeki *et al.*, 2004
pUC57	XBP1-XTEN	This study
p415GPDp	pGPDp-Rqc2-FLAG-TEV-ProteinA	Matsuo *et al.*,2020
pCMV-VSV-G-RSV-Rev		RIKEN BioResource Center (RDB04393)
pCAG-HIVgp		RIKEN BioResource Center (RDB04394)
CSII-CMV-MCS-IRES2-Bsd		RIKEN BioResource Center (RDB04385)
CSII-CMV-MCS-IRES2-Bsd	kozak-human eS10-3HA	This study
CSII-CMV-MCS-IRES2-Bsd	kozak-human eS10(K138R/K139R)-3HA	This study
CSII-CMV-MCS-IRES2-Bsd	kozak-human uS10-3HA	This study
CSII-CMV-MCS-IRES2-Bsd	kozak-human uS10(K4R/K8R)-3HA	This study

Supplementary Table 2: plasmids list

Parent strain	Genotype	Reference
HEK293T	Wild-type	RIKEN BioResource Center (RCB2202)
Lenti-X 293T	shRNA non-silencing	Hashimoto *et al.*, 2020; Lenti-X 293T (Takarabio, Z2180N)
Lenti-X 293T	shRNA against ASCC1	Hashimoto *et al*., 2020; Lenti-X 293T (Takarabio, Z2180N)
293FT		ThermoFisher (R700-07)

Supplementary Table 3: cell lines list

Antibodies	Source	Catalog number	The dilution used for Western blotting
Anti-HA-Peroxidase	Roche	Cat# 12013819001, RRID:AB_390917	1:5000
Anti-FLAG M2 antibody	Sigma	Cat# F1804-1MG	1:5000
Anti-Ubiquitin (P4D1) HRP	Santa Cruz Biotechnology	Cat# sc-8017	1:1000
Anti-PA-Peroxidase	Wako	Cat# 015-25951	1:5000
Anti-GAPDH	Wako	Cat# 016-25523; RRID: AB_2814991	1:5000
Anti-eS10 antibody	Abcam	Cat# ab151550; RRID: AB_2714147	1:1000
Anti-uS10 antibody	Abcam	Cat# ab133776	1:1000
Anti-ASCC3 antibody	Proteintech	Cat# 17627-1-AP-150	1:1000
Anti-ASCC2 antibody	Bethyl Labs	Cat# A304-020A	1:1000
Anti-ASCC1 antibody	Bethyl Labs	Cat# A303-871A	1:1000
Anti-TRIP4 antibody	Bethyl Labs	Cat# A300-843A	1:1000
Anti-Ubiquitin, Lys63-Specific(Apu3) antibody	Millipore	Cat# 05-1308	1:1000
Anti-p38MAPK antibody	Cell Signaling Technology	Cat# 8690	1:2000
Anti-phospho-p38MAPK(Thr180/Tyr182) antibod	Cell Signaling Technology	Cat# 9211	1:2000
Anti-phospho-JNK antibody (Thr183/Tyr185)	Cell Signaling Technology	Cat# 4668	1:2000
ECL Anti-mouse IgG, horseradish Peroxidase	GE Healthcare	Cat# NA931V	1:5000
ECL Anti-rabbit IgG, horseradish Peroxidase	GE Healthcare	Cat# NA934V	1:5000

Supplementary Table 4: antibodies list

Aim	Primer ID	Sequence (5' to 3')
Template DNA for *XBP1u* mRNAs	XBP1u_Fw	GGCCAGTGAATTCTAATACGACTCACTATAGGGACACTTGCTTTTGACACAACAGCCACCATGCATCATCATCATCATCATGGTGTTGCTATGCCAGGTGCTGAAGATGATGTTGTTGGAGTGGTGGTGGCAGCC
	XBP1u_V193-Stop_Rev	TACGCCCTCGAGACGTGCGCAGCTGGCCACGTACGTATCGCTATCTAGCTATACCGCCAGAATCCATGGG
	XBP1u(delta236-261)_Rev	TACGCCCTCGAGACGTGCGCAGCTGGCCACGTACGTATCGCTATCTAGCTACTGGGTAGACCTCTGGGAGCTCCT
	XBP1u_V193-(194-261)_Rev	TACGCCCTCGAGACGTGCGCAGCTGGCCACGTACGTATCGCTATCTAGCTAGTTCATTAATGGCTTCCAGCTTG
	XBP1u_V193-(194-261)_W256A Rev	TACGCCCTCGAGACGTGCGCAGCTGGCCACGTACGTATCGCTATCTAGCTAGTTCATTAATGGCTTTGCGCTTG
	XBP1u_V193-polyA60_Rev	TACGCCCTCGAGACGTGCGCAGCTGGCCACGTACGTATCGCTATCTAGCTATTTTTTTTTTTTTTTTTTTTTTTTTTTTTTTTTTTTTTTTTTTTTTTTTTTTTTTTTTTTTACCGCCAGAATCCATGGGGA
	XBP1u_V193-stemloop_Rev	TACGCCCTCGAGAACGTGCGCAGCTGGCCACGTACGTATCGCTATCTAGCTACGATATCCCGTGGAGGGGCGCGTGGTGGCGGCTGCAGCCGCCACCACGCGCCCCTCCACGGGATATCGTACCGCCAGAATCCATGGGGA
	XBP1u_V193-truncated_Rev	TACCGCCAGAATCCATGGG
	XBP1u_V193-Ribozyme_Rev	TACGCCCTCGAGCGTGCGCAGCTGGCCACGTACGTATCGCTATCTAGCTACCTGTTTCGTCCTCACGGACTCATCAGACCGGAAAACACATCCGGTGACAGGTACC GCCAGAATCCATGGGGA
Template DNA for *XBP1*-XTEN mRNA	Xbp1-XTEN-Fw	GGGATGAACTAATACGACTCACTATAGGCCCTCC
	Xbp1-XTEN-Rv	TTAAAAAGAAAGGCCTTACATATTTATTACTGAATCCAGCCAACC

Supplementary Table 5: primer list

Supplementary References

1. Wu, C.C., et al., Ribosome Collisions Trigger General Stress Responses to Regulate Cell Fate. *Cell*, 2020. **182**(2): p. 404-416.e14.
2. Schellenberger, V., et al., A recombinant polypeptide extends the in vivo half-life of peptides and proteins in a tunable manner. *Nat Biotechnol*, 2009. **27**(12): p. 1186-90.
3. Punjani, A., et al., cryoSPARC: algorithms for rapid unsupervised cryo-EM structure determination. *Nat Methods*, 2017. **14**(3): p. 290-296.
4. Goddard, T.D., et al., UCSF ChimeraX: Meeting modern challenges in visualization and analysis. *Protein Sci*, 2018. **27**(1): p. 14-25.
5. Kerpedjiev, P., S. Hammer, and I.L. Hofacker, Forna (force-directed RNA): Simple and effective online RNA secondary structure diagrams. *Bioinformatics*, 2015. **31**(20): p. 3377-9.
