# Supplementary material for: A distinct mammalian disome collision interface harbors K63-linked polyubiquitination of uS10 to trigger hRQT-mediated subunit dissociation
Source: Nat Commun. 2022 Oct 27;13:6411. doi: 10.1038/s41467-022-34097-9 (PMC9613687; doi:10.1038/s41467-022-34097-9)
Supplement: Supplementary file 3 — Reporting Summary [file 41467_2022_34097_MOESM3_ESM.pdf]

nature portfolio

Corresponding author(s): Toshifumi Inada

Last updated by author(s): Sep 5, 2022

Reporting Summary

Nature Portfolio wishes to improve the reproducibility of the work that we publish. This form provides structure for consistency and transparency in reporting. For further information on Nature Portfolio policies, see our Editorial Policies and the Editorial Policy Checklist.

Statistics

For all statistical analyses, confirm that the following items are present in the figure legend, table legend, main text, or Methods section.

n/a	Confirmed	
☐	☒	The exact sample size (n) for each experimental group/condition, given as a discrete number and unit of measurement
☐	☒	A statement on whether measurements were taken from distinct samples or whether the same sample was measured repeatedly
☒	☐	The statistical test(s) used AND whether they are one- or two-sided<br>*Only common tests should be described solely by name; describe more complex techniques in the Methods section.*
☒	☐	A description of all covariates tested
☒	☐	A description of any assumptions or corrections, such as tests of normality and adjustment for multiple comparisons
☐	☒	A full description of the statistical parameters including central tendency (e.g. means) or other basic estimates (e.g. regression coefficient) AND variation (e.g. standard deviation) or associated estimates of uncertainty (e.g. confidence intervals)
☒	☐	For null hypothesis testing, the test statistic (e.g. F, t, r) with confidence intervals, effect sizes, degrees of freedom and P value noted<br>*Give P values as exact values whenever suitable.*
☒	☐	For Bayesian analysis, information on the choice of priors and Markov chain Monte Carlo settings
☒	☐	For hierarchical and complex designs, identification of the appropriate level for tests and full reporting of outcomes
☒	☐	Estimates of effect sizes (e.g. Cohen's d, Pearson's r), indicating how they were calculated

Our web collection on statistics for biologists contains articles on many of the points above.

Software and code

Policy information about availability of computer code

Data collection	EPU 2.12.1
Data analysis	Cryo-EM data were processed using MotionCor2 1.4.0, Gctf 1.06, Gautomatch v0.56, cryoSPARC 3.2.0 and Relion 3.1.3. Molecular models were built and refined using Coot 0.9 and Phenix 1.19. Structural figures were created using ChimeraX 1.3.

For manuscripts utilizing custom algorithms or software that are central to the research but not yet described in published literature, software must be made available to editors and reviewers. We strongly encourage code deposition in a community repository (e.g. GitHub). See the Nature Portfolio guidelines for submitting code & software for further information.

Data

Policy information about availability of data

All manuscripts must include a data availability statement. This statement should provide the following information, where applicable:

- Accession codes, unique identifiers, or web links for publicly available datasets
- A description of any restrictions on data availability
- For clinical datasets or third party data, please ensure that the statement adheres to our policy

The cryo-EM structural data generated in this study have been deposited in the Protein Data Bank and in the Electron Microscopy Data Bank databases under accession codes PDB: 7QVP [https://doi.org/10.2210/pdb7QVP/pdb] and EMDB-14181 [https://www.ebi.ac.uk/emdb/EMD-14181].

Field-specific reporting

Please select the one below that is the best fit for your research. If you are not sure, read the appropriate sections before making your selection.

☒ Life sciences ☐ Behavioural & social sciences ☐ Ecological, evolutionary & environmental sciences

For a reference copy of the document with all sections, see nature.com/documents/nr-reporting-summary-flat.pdf

Life sciences study design

All studies must disclose on these points even when the disclosure is negative.

Sample size	All experiments were repeated at least two times independently and s.e.m were calculated from those data. We described in the figure legends.
Data exclusions	No data were excluded intentionally.
Replication	For each series of experiments, all replication attempts were successful.
Randomization	No randomrization.
Blinding	The investigators were not blinded during data collection.

Reporting for specific materials, systems and methods

We require information from authors about some types of materials, experimental systems and methods used in many studies. Here, indicate whether each material, system or method listed is relevant to your study. If you are not sure if a list item applies to your research, read the appropriate section before selecting a response.

Materials & experimental systems

n/a	Involved in the study	
☐	☒	Antibodies
☐	☒	Eukaryotic cell lines
☒	☐	Palaeontology and archaeology
☒	☐	Animals and other organisms
☒	☐	Human research participants
☒	☐	Clinical data
☒	☐	Dual use research of concern

Methods

n/a	Involved in the study	
☒	☐	ChIP-seq
☒	☐	Flow cytometry
☒	☐	MRI-based neuroimaging

Antibodies

Antibodies used

Anti-HA-Peroxidase, Roche Cat# 12013819001, RRID:AB_390917
Anti-FLAG M2 antibody, Sigma Cat# F1804-1MG
Anti-Ubiquitin (P4D1) HRP, Santa Cruz Biotechnology Cat# sc-8017
Anti-Ubiquitin, Lys63-Specific(Apu3) antibody, Millipore Cat# 05-1308
Anti-PA-Peroxidase, Wako Cat# 015-25951
Anti-GAPDH, Wako Cat# 016-25523; RRID: AB_2814991
Anti-eS10 antibody, Abcam Cat# ab151550; RRID: AB_2714147
Anti-uS10 antibody, Abcam Cat# ab133776
Anti-ASCC3 antibody, Proteintech Cat# 17627-1-AP-150
Anti-ASCC2 antibody, Bethyl Labs Cat# A304-020A
Anti-ASCC1 antibody, Bethyl Labs Cat# A303-871A
Anti-TRIP4 antibody, Bethyl Labs Cat# A300-843A
Anti-p38MAPK antibody, Cell Signaling Technology Cat# 8690
Anti-phospho-p38MAPK(Thr180/Tyr182) antibody, Cell Signaling Technology Cat# 9211
Anti-phospho-JNK antibody (Thr183/Tyr185), Cell Signaling Technology Cat# 4668 ECL
Anti-mouse IgG, horseradish Peroxidase GE Healthcare Cat# NA931V ECL
Anti-rabbit IgG, horseradish Peroxidase GE Healthcare Cat# NA934V

Validation	We used following antibodies for western blotting; For peptidyl-tRNA derived from in vitro translation (Fig 1c-e; Fig S6c-d), Anti-PA-Peroxidase (Wako Cat# 015-25951; 1:5000); For the detection of the (non-)ubiquitinated uS10 (Fig 1f; Fig 4c-f; Fig5 c, f, g; Fig 6 a-d; Fig S6, c-d), Anti-uS10 antibody (Abcam Cat# ab133776; 1:1000); For the detection of the (non-)ubiquitinated eS10 (Fig 1f; Fig 4c-f; Fig5 c, f, g; Fig 6 a-d; Fig S6, c-d), Anti-eS10 antibody (Abcam Cat# ab151550; RRID: AB_2714147; 1:1000); For the detection of 3FLAG-ZNF598 (Fig 1f) and 3FLAG-ASCC3 (Fig 4f; Fig 6b, d), Anti-FLAG M2 antibody (Sigma Cat# F1804-1MG; 1:5000); For the detection of mono-ubiquitin or poly-ubiquitin formed by Mms2-Ubc13 dependent reaction (Fig 5 d-e), Anti-Ubiquitin (P4D1) HRP (Santa Cruz Biotechnology Cat# sc-8017; 1:1000); For the detection of K63-linked polyubiquitin chain formed by Mms2-Ubc13 (Fig S7d), Anti-Ubiquitin, Lys63-Specific(Apu3) antibody (Millipore Cat# 05-1308; 1:1000); For the detection of 3FLAG-ASCC3 of the purified hRQT complex (Fig 4d; Fig 5d-g; Fig S6d; Fig S7a-b), Anti-ASCC3 antibody (Proteintech Cat# 17627-1-AP-150; 1:1000); For the detection of HA-ASCC2 of the purified hRQT complex (Fig 5d-e; Fig S7a-b), Anti-ASCC2 antibody (Bethyl Labs Cat# A304-020A; 1:1000); For the detection of HA-TRIP4 of the purified hRQT complex (Fig 5d-e; Fig S7a-b), Anti-TRIP4 antibody (Bethyl Labs Cat# A300-843A; 1:1000); For the detection of ASCC1 of the purified hRQT complex without treatment by shRNA against ASCC1 (Fig S7a), Anti-ASCC1 antibody (Bethyl Labs Cat# A303-871A; 1:1000); For the detection of HA-ASSC2, HA-TRIP4 of the purified hRQT complex (Fig 4d, f; Fig 5f-g; Fig S6d; Fig S7a-b) and the detection of uS10-3HA or eS10-3HA (Fig S1b-c), Anti-HA-Peroxidase (Roche Cat# 12013819001, RRID:AB_390917; 1:5000); For the detection of p38 after the treatment with anisomycin (Fig S1b-c), Anti-p38MAPK antibody (Cell Signaling Technology Cat# 8690; 1:2000); For the detection of phospho-p38MAPK after the treatment with anisomycin (Fig S1b-c), Anti-phospho-p38MAPK (Thr180/Tyr182) antibody (Cell Signaling Technology Cat# 9211; 1:2000); For the detection of phospho-JNK after the treatment with anisomycin (Fig S1b-c), Anti-phospho-JNK antibody (Thr183/Tyr185) (Cell Signaling Technology Cat# 4668 ECL; 1:2000). For the detection of GAPDH (Fig S1c, d), Anti-GAPDH Wako (Cat# 016-25523; RRID: AB_2814991; 1:5000).<br>After incubation in primary antibodies, sections were washed in PBS and incubated with conjugated secondary antibodies, Anti-mouse IgG, horseradish Peroxidase (GE Healthcare Cat# NA931V ECL; 1:5000) or Anti-rabbit IgG, horseradish Peroxidase (GE Healthcare Cat# NA934V; 1:5000).

Eukaryotic cell lines

Policy information about cell lines

Cell line source(s)	HEK293T (RIKEN BioResource Center), Lenti-X 293T(Takarabio, Z2180N) expressing control shRNA or shRNA agasinst ASCC1 (Hashimoto et al., doi:10.1038/s41598-020-60241-w, established in Tohoku University) , 293FT (ThermoFisher Scientific)
Authentication	HEK293T (RCB2202), Lenti-X 293T (Takarabio, Z2180N) expressing control shRNA or shRNA against ASCC1 (Hashimoto et al., doi:10.1038/s41598-020-60241-w, established in Tohoku University), 293FT (R70007)
Mycoplasma contamination	Mycoplasma contamination was not detected with DAPI staining.
Commonly misidentified lines (See ICLAC register)	No commonly misidentified cell lines were used in the study
